# In Vitro Studies Regarding the Safety of Chitosan and Hyaluronic Acid-Based Nanohydrogels Containing Contrast Agents for Magnetic Resonance Imaging

**DOI:** 10.3390/ijms23063258

**Published:** 2022-03-17

**Authors:** Cecilia Virginia Gheran, Sorina Nicoleta Voicu, Bianca Galateanu, Maité Callewaert, Juliette Moreau, Cyril Cadiou, Françoise Chuburu, Anca Dinischiotu

**Affiliations:** 1 Department of Biochemistry and Molecular Biology, Faculty of Biology, University of Bucharest, 050095 Bucharest, Romania; virginia.gheran@gmail.com (C.V.G.); bianca.galateanu@bio.unibuc.ro (B.G.); anca.dinischiotu@bio.unibuc.ro (A.D.); 2 Institut de Chimie Moléculaire de Reims, CNRS UMR 7312, Université de Reims Champagne-Ardenne URCA, CEDEX 2, F-51685 Reims, France; maite.callewaert@univ-reims.fr (M.C.); juliette.moreau@univ-reims.fr (J.M.); cyril.cadiou@univ-reims.fr (C.C.); francoise.chuburu@univ-reims.fr (F.C.)

**Keywords:** nanohydrogel, chitosan, contrast agents, RAW 264.7 cell line, biocompatibility, hemocompatibility

## Abstract

The aim of this study was to investigate the biocompatibility of contrast agents, such as gadolinium 1, 4, 7, 10 tetraazacyclo-dodecane tetraacetic acid (GdDOTA) and gadolinium dioctyl terephthalate (GdDOTP), encapsulated in a polymeric matrix containing chitosan and hyaluronic acid using RAW264.7 murine macrophages and human blood samples. The cell viability and cytotoxicity were evaluated by 3-(4,5-dimethylthiazol-2-yl)-2,5-diphenyltetrazolium bromide (MTT) and lactate dehydrogenase (LDH) assays, while cell cycle analysis was determined in RAW264.7 cells using flow cytometry. The mitochondrial membrane potential (MMP), hemolytic index, complement activation, and thrombogenic potential of gadolinium (Gd) containing nanohydrogels were measured by fluorometric and spectrophotometric methods. Taken together, our results demonstrate the good bio- and hemocompatibility of chitosan-based nanohydrogels with the RAW264.7 cell line and human blood cells, suggesting that these could be used as injectable formulations for the magnetic resonance imaging diagnostic of lymph nodes.

## 1. Introduction

Cancer, the second leading cause of death worldwide, is a very important issue in the health care field.

In many cancers, the lymphatic system, as an essential part of the immune system comprising a network of lymph vessels, organs, and lymph nodes, is a major route in the dissemination of metastatic cancer cells.

Large-scale clinical trials have shown significant differences in the morbidity and mortality of patients with occult micro metastases (lymph node metastases less than 2 mm in diameter) at the time of diagnosis [1,2].

Detection of a single lymph node metastasis can be correlated with a poor prognosis of patients by approximately 50%, regardless of the location or size of the primary tumor [3,4,5].

Therefore, for cancer patients, in addition to histopathology and the description of tumor size, the identification of metastases in the lymph nodes (local and distant) plays a particularly important role from a clinical point of view in the prognosis of malignancy and establishment of an appropriate treatment [5,6].

As alternatives for assessing lymph node status, non-invasive imaging techniques including computed tomography (CT), nuclear magnetic resonance imaging (MRI), and ultrasonography (US) have been developed and are routinely used in clinical practice to diagnose metastases in lymph nodes [7,8,9].

Relying only on morphological criteria (shape, number, density, dynamic contrast) and the size of lymph nodes (>10 mm) in differentiating between benign and malignant structures, these imaging tools suffer from a lack of sensitivity (45–90%) [1,10].

However, MRI seems to be more attractive for investigating the lymphatic system because of its advantages consisting in excellent 3D spatial resolution, non-ionizing radiation, non-invasiveness, and infinite tissue penetration [11,12,13]. Furthermore, its sensitivity and specificity could be improved by the interstitial (intracutaneous or subcutaneous) or intravenous injection of two categories of contrast agents with specific lymph node accumulation, including extracellular gadolinium chelates [14,15] and superparamagnetic iron oxide nanoparticles (SPION) [16,17] as positive and negative ones, according to the contrast they provide on the images.

Among these contrast agents, extracellular gadolinium chelates are the most commonly used, being administered via the interstitial route for lymph node imaging. Gadolinium-based contrast agents (GBCA) present Gd^3+^ bound tightly by an organic ligand in order to diminish the possible toxicity of the free ion and improve their pharmacologic properties. These could be linear or cyclic, charged or nonionic [18]. The macrocyclic and ionic structures, such as DOTA and DOTP, are more thermodynamically stable than linear and nonionic ones and less toxic [19]. MR lymphography has been reported either on animal models [20,21] or in human volunteers [22,23,24]. Because of their low molecular weights, these lymphotropic agents diffuse into the blood system, with only a small amount of the injected dose reaching the lymphatic system [25,26,27]. Moreover, they have shown a poor accumulation in the regional lymph nodes and have been rapidly cleared [28]. Thus, higher doses and injection volumes were needed for administration, which could be problematic for patients with kidney diseases [29,30,31]. Persons with acute kidney disease could develop nephrogenic systemic fibrosis (NFS) caused by GBCA [32] since these are excreted predominantly in urine by glomerular filtration. NFS is a debilitating and sometimes fatal systemic condition characterized by skin and subcutaneous abnormalities triggered by GBCA, the mechanism of which is still poorly understood [33]. However, according to several physicians, the practical use of macrocyclic GBCA in patients with acute kidney injury and/or severe chronic kidney disease significantly diminished the NFS cases [34].

To enhance MRI sensitivity and reduce the injected gadolinium dose, different nano-sized agents integrating gadolinium chelates, such as nanogels [35,36], micelles [37], dendrimers [38], fullerenes [39], as well as polymeric [40], lipid-based [41], and silica nanoparticles [42], have been designed.

As long as these vectors are developed for in vivo administration, their biocompatibility analysis is mandatory.

Natural polysaccharides are good candidates in the biomedical field [43,44] due to their water-solubility, hydrophilicity, and biodegradability, and among them chitosan (CS) and hyaluronic acid (HA) are probably the most currently used [45,46,47,48,49,50,51].

Chitosan is a cationic polysaccharide constituted of β-(1−4)-linked D-glucosamine and N-acetyl-D glucosamine residues and produced by the deacetylation of chitin (poly-β-(1→4)- N-acetyl-D-glucosamine) under alkaline conditions [52]. Due to its cationic character, chitosan exhibits antimicrobial [53], antioxidant [54], and antitumor [55] properties, as well as muco-adhesiveness [56] and an ability to open epithelial tight junctions, thus allowing the transport of drug across cellular barriers.

For these features, chitosan and its derivatives are used in a wide range of biomedical and pharmaceutical applications, such as drug delivery systems [57,58,59,60], tissue engineering [61,62], as well as bioimaging and gene therapy [63,64].

Over the past few years, we have worked on the development of formulations based on ionic gelation between chitosan and hyaluronic acid, to produce nanogels as MRI contrast agents. We have proven that the encapsulation of GBCA in these biopolymer nanoparticles significantly increases the efficiency of MRI signal [65]. This improvement has been explained due to the confinement of contrast agents within the polymeric matrix, which induces a high local concentration of Gd, and to the confinement of water molecules through the gelled network, as well as to the establishment of an extended network of H-bonding interactions between Gd chelates and surrounding water molecules [66].

The aim of this study was to go further in the precise characterization of these systems and particularly to investigate the biocompatibility, mitochondrial membrane potential, hemolytic index, complement activation, and thrombogenic potential of these nanogels that incorporated GdDOTA and GdDOTP as MRI contrast agents.

## 2. Results

### 2.1. Physico-Chemical Characteristics of GdDOTA (GdDOTA⊂CS-TPP/HA) and GdDOTP (GdDOTP⊂CS-TPP/HA) Nanohydrogels

Nanogels (NGs) were obtained thanks to an ionotropic gelation process [65,66]. For that purpose, chitosan was solubilized in an acidic solution and was allowed to react with polyanions (HA and sodium tripolyphosphate TPP), leading to the formation of polyelectrolyte complexes through multivalent electrostatic interactions. The encapsulation of Gd-based contrast agents was conducted in the same way. The complexes GdDOTA or GdDOTP were thus dissolved in the polyanionic phase before the formation of the nanoparticles. Stable colloidal nanosuspensions were obtained and, after a step of dialysis that removes unreacted compounds and raises the pH, GdDOTA⊂CS-TPP/HA and GdDOTP⊂CS-TPP/HA NGs were characterized. Physicochemical properties were evaluated by dynamic light scattering (DLS) and electrophoretic light scattering (ELS) while the gadolinium concentrations within the purified nanogels were determined by inductively coupled plasma (ICP) titrations (Table 1).

For both formulations, average diameters of nanogels were found to be compatible with in vivo injections. The low PdI values indicated that the nanosuspensions were monodispersed. The high ζ-potential values ensured a good stability of the suspensions, thanks to efficient electrostatic repulsions between particles. Moreover, high concentrations of gadolinium were determined by ICP titrations for GdDOTA⊂CS-TPP/HA and GdDOTP⊂CS-TPP/HA nanogels, which highlighted the efficacy of nanogels to behave as good cargos for Gd-based contrast agents.

### 2.2. Biological Assays

In order to evaluate the biological effects induced by lymphotropic nanohydrogels (NGs), the viability, cytotoxicity, hemolytic index, thrombogenic potential, and complement activation were investigated. In addition, cell cycle distribution and the mitochondria membrane potential were analyzed.

**Figure 1:**
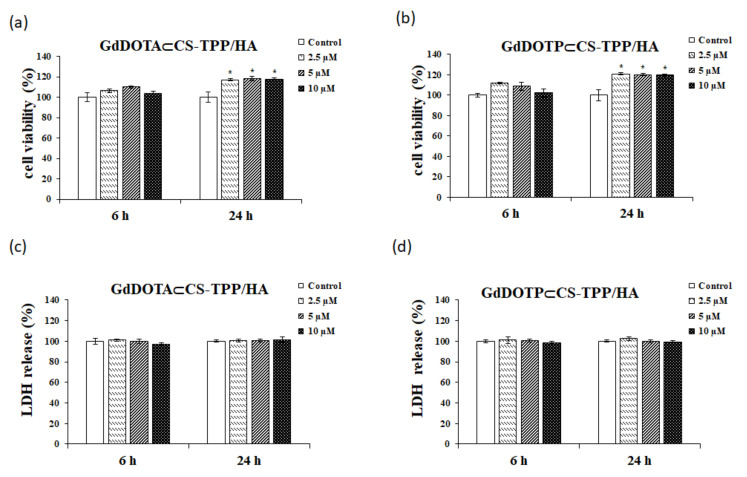
The MTT (**a**,**b**) and LDH (**c**,**d**) assays in presence of RAW 264.7 cells after 6- and 24 h exposed to doses of 2.5, 5 and 10 μM of (**a**,**c**) GdDOTA⊂CS-TPP/HA and (**b**,**d**) GdDOTP⊂CS-TPP/HA NGs. Untreated cells were used as a control. Data are expressed as mean ± SD (*n* = 3). The asterisks represent the statistical significance obtained by the Student’s test, as follows: * *p* < 0.05 (significant).

The viability test for RAW 264.7 murine macrophages was performed by the 3-(4, 5-dimethylthiazol-2-yl)-2, 5-diphenyltetrazolium bromide (MTT) assay. RAW 264.7 cells were treated for 6 and 24 h, respectively, with GdDOTA⊂CS-TPP/HA and GdDOTP⊂CS-TPP/HA at doses of 2.5, 5, and 10 μM. As shown in Figure 1a,b, regardless of the interval treatment and dose applied, the nanohydrogels did not induce toxic effects in the RAW 264.7 cell line. Moreover, after the 24-h interval, significant increases in cell viability were registered, reaching an increase of 18% for GdDOTA⊂CS-TPP/HA and 20% for GdDOTP⊂CS-TPP/HA, respectively, compared to control.

The integrity of the cell membrane was evaluated by the LDH test (Figure 1c,d). The data obtained show that, throughout the treatment duration (24 h), both GdDOTA⊂CS-TPP/HA and GdDOTP⊂CS-TPP/HA at doses between 2.5 and 10 μM did not induce significant changes in this parameter compared to untreated cells. These results suggest the absence of cytotoxic potential for these formulations.

### 2.3. Mitochondrial Membrane Potential (MMP) Assessment

**Figure 2:**
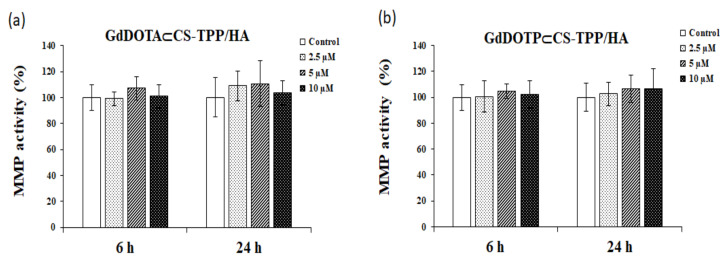
The level of mitochondrial membrane potential in RAW 264.7 murine macrophages cells after 6 -and 24 h of exposure to doses of 2.5, 5 and 10 μM of (**a**) GdDOTA⊂CS-TPP/HA and (**b**) GdDOTP⊂CS-TPP/HA NGs. Untreated cells were used as a control. Data are expressed as mean ± SD (*n* = 3).

The evaluation of the mitochondrial function of RAW 264.7 macrophages exposed to GdDOTA⊂CS-TPP/HA and GdDOTP⊂CS-TPP/HA was performed by testing the mitochondrial membrane potential (MMP) using a fluorescent cationic carbocyanine dye (JC-1) (Figure 2).

After 24 h of exposure of RAW 264.7 cells at high doses (2.5, 5, and 10 μM) of GdCA⊂CS-TPP/HA (GdCA = GdDOTA, GdDOTP), no decrease of the mitochondrial membrane potential compared to untreated cells was noticed.

### 2.4. RAW 267.4 Cell Cycle Distribution after GdDOTA⊂CS-TPP/HA and GdDOTP⊂CS-TPP/HA NGs Treatment

**Figure 3:**
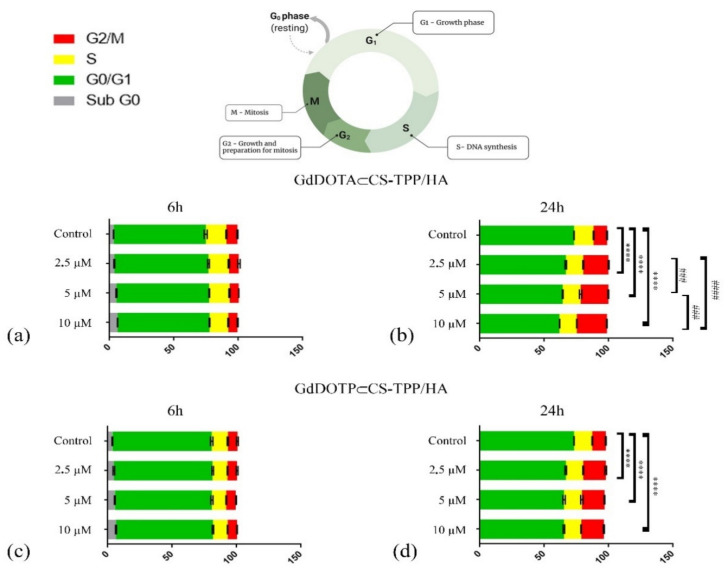
Cell cycle distribution in RAW 267.4 murine macrophage cells treated with (**a**) GdDOTA⊂CS-TPP/HA NGs for 6 h; (**b**) GdDOTA⊂CS-TPP/HA NGs for 24 h: **** *p* < 0.0001 control versus 2.5 µM_G0/G1 and G2/M; control versus 5 µM_G0/G1 and G2/M and control versus 10 µM_G0/G1 and G2/M); ### *p* < 0.001 2.5 µM versus 5 µM _G0/G1 and G2/M and 5 µM versus 10 µM _G0/G1 and G2/M; #### *p* < 0.0001 2.5 µM versus 10 µM _G0/G1 and G2/M; (**c**) GdDOTP⊂CS-TPP/HA NGs for 6 h and (**d**) GdDOTP⊂CS-TPP/HA NGs for 24 h: **** *p* < 0.0001 control versus 2.5 µM_G0/G1 and G2/M; control versus 5 µM_G0/G1 and G2/M and control versus 10 µM_G0/G1 and G2/M.

As shown in Figure 3a, c, RAW 267.4 cell cycle distribution was not influenced after 6 h of treatment with GdDOTA⊂CS-TPP/HA and GdDOTP⊂CS-TPP/HA NGs as compared with the untreated samples. 

However, both GdDOTA⊂CS-TPP/HA and GdDOTP⊂CS-TPP/HA NGs treatments induced a significant increase of RAW 267.4 cell proliferation after 24 h of treatment as compared with 6 h of treatment (**** *p* < 0.0001). As shown in Figure 3b,d, at 24 h of treatment, GdDOTA⊂CS-TPP/HA NGs induced a significant dose-dependent increase of G2/M cells, while the GdDOTP⊂CS-TPP/HA NGs concentration did not influence the cell cycle distribution. Interestingly, after 6 h of treatment with both GdDOTA⊂CS-TPP/HA and GdDOTP⊂CS-TPP/HA NPs, flow cytometry histograms revealed a small sub-G0 population of RAW 267.4 cells that disappeared after 24 h, as a proof of the treatment’s nontoxic character at all applied doses.

### 2.5. Evaluation of Complement Activation by the GdDOTA⊂CS-TPP/HA and GdDOTP⊂CS-TPP/HA NGs

**Figure 4:**
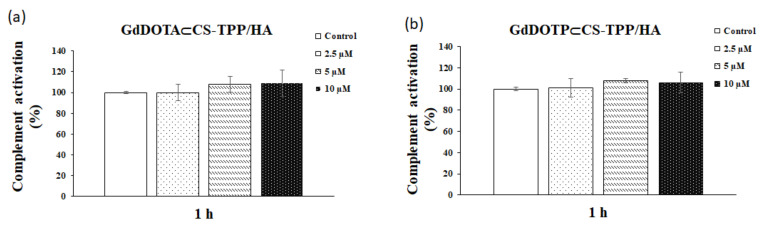
Classical activation of the complement system after 1 h of exposure to doses of 2.5, 5 and 10 μM of (**a**) GdDOTA⊂CS-TPP/HA and (**b**) GdDOTP⊂CS-TPP/HA NGs. A human serum sample incubated at 37 °C served as a negative (normal) control. Data are expressed as mean ± SD (*n* = 3).

Figure 4a,b shows that, after one hour of incubation of human serum samples with GdDOTA⊂CS-TPP/HA and GdDOTP⊂CS-TPP/HA NGs in doses of 2.5, 5, and 10 µM, no significant changes were noticed compared with negative control. 

### 2.6. Hemolytic Activity of the GdDOTA⊂CS-TPP/HA and GdDOTP⊂CS-TPP/HA NGs

**Figure 5:**
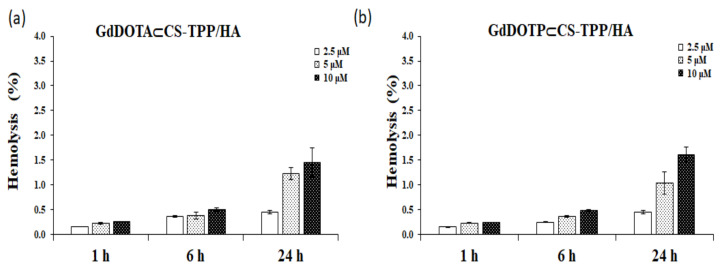
The percentage of hemolysis after 1, 6 and 24 h of incubation with (**a**) GdDOTA⊂CS-TPP/HA and (**b**) GdDOTP⊂CS-TPP/HA NGs, at doses of 2.5, 5 and 10 μM. Data are expressed as mean ± SD (*n* = 3). As a positive (100% hemolysis) and negative (0% hemolysis) controls, supernatants resulting from red blood cells treated with Triton X-100 1% and physiological serum (0.9% NaCl) respectively have been used.

As shown in Figure 5a,b, the percentage of hemolytic activity for both types of nanohydrogels was less than 5% (accepted standard limit, according to the ISO 10993-4), regardless on the time and dose tested.

The maximum hemolysis rates induced after the 24-h interval were 1.45% ± 0.29% for GdDOTA⊂CS TPP/HA and 1.61% ± 0.15% for GdDOTP⊂CS-TPP/HA, respectively. Thus, both GdDOTA⊂CS TPP/HA and GdDOTP⊂CS-TPP/HA NGs have been shown to be hemocompatible. 

### 2.7. Evaluation of the Thrombogenic Potential of Gd Nanohydrogels

In order to evaluate the thrombogenic potential of nanohydrogels, human whole blood was allowed to coagulate in contact with the GdDOTA⊂CS-TPP/HA and GdDOTP⊂CS-TPP/HA NGs at doses of 2.5, 5, and 10 μM for 5, 15, 25, and 35 min. 

The results of thrombogenic activity are shown in Table 2. As the coagulation process progresses, more erythrocytes are retained in the clot, which involves the release of a smaller amount of hemoglobin by lysis upon the addition of distilled water. 

Analysis of Table 2 reveals that both GdDOTA⊂CS-TPP/HA and GdDOTP⊂CS-TPP/HA presented a similar behavior compared to the negative control (PBS), and could thus be classified as non-thrombogenic NGs.

## 3. Discussion

The design strategy of lymphotropic nanohydrogels by encapsulating known contrast agents, namely GdDOTA and GdDOTP, in a biocompatible hydrophilic polymeric matrix composed of chitosan and hyaluronic acid (which are the subject of this study) was proposed from the perspective of NSF risk, especially in patients with renal impairment, in order to improve their ability to relax and thus reduce the doses required for administration.

Macrophages are important in immune and inflammatory responses and are involved in the early events in various fibrotic processes [67]. Moreover, several studies suggest that these could play a pivotal role in NSF [68]. They are also used as cell models in a series of in vitro studies for the evaluation of the potential cytotoxic effects of GBCA [69,70].

Most of the studies that investigated GBCA toxicity have focused on changes induced by concentrations higher than 0.5 mM, which does not exactly reflect human physiological conditions and might not be directly applicable to the pathogenesis of NSF or the deposition of this lanthanide in the brain level [71]. Moreover, for people with normal renal function, the mean half-life by elimination of GBCA is approximately 1.2 h, which indicates that very low doses and traces of GBCA may interact with macrophages [72].

Following these issues, a recent study led by Weng et al. [68] aimed to evaluate the immune response generated by RAW 264.7 cells by exposing them for 24 h to low doses (0.25–2.5 μM) of different types of GBCA (Primovist^®^, Omniscan^®^, Magnevist^®^, Gadovist^®^) and GdCl_3_ [68]. During the experiment, none of the GBCA induced negative effects on the growth of RAW 264.7 cells. Another in vitro study on RAW 264.7 cell line was conducted by Jesus et al., in order to evaluate the immunotoxicity profile of chitosan nanoparticles and polymers (deacetylation degree of 80% and 93%) using the MTT metabolic activity assay [73].

According to our data, cell viability increased after 24 h treatment of RAW 264.7 cells with GdDOTA⊂CS-TPP/HA and GdDOTP⊂CS-TPP/HA, which could be correlated with the results of cell cycle analyses. This could be due to the fact that chitosan exposure increased the number of intercellular junctions (RAW 264.7 cells being adherent) and induced significant proliferation, as has previously been proven on 3T3 mouse fibroblasts [74]. This could be due to the presence of natural polymers, which could promote cell proliferation [75].

Similar results have been obtained with albumin-based nanoparticles loaded with hydrophobic gadolinium chelates (GGD-BSA) as contrast agents for liver tumors imaging, using RAW 264.7 and human hepatocellular carcinoma cells [76], as well as with hyaluronic acid and chitosan hydrogels (CS-HA) designed for therapeutic angiogenesis, tested on rat adipose tissue isolated (rASC) and HUVEC cells [77].

The depolarization of mitochondrial membrane potential (ΔΨm) is a well-known indicator of cellular toxicity [78], and the accumulation of ROS is considered an essential initiator of ΔΨm decrease, involving the impairment of mitochondrial function [79].

Our experimental data proved that cells’ exposure to GdDOTA⊂CS TPP/HA and GdDOTP⊂CS-TPP/HA NGs did not alter the mitochondrial membrane potential. This could be explained based on our previous results that proved that these NGs did not generate ROS in SVEC cells [51].

Other studies have also shown that MMP integrity is maintained by the interaction of cells with different types of nanoparticles [80,81]. The potential of the inner mitochondrial membrane plays an important role in the oxidative phosphorylation, which is based on the proton gradient generated by the reoxidation of NADH and FADH2 in the electron transfer chain of mitochondria. A dysfunction of this can increase electron leakage and the generation of ROS in cells. In our experimental conditions, gadolinium nanohydrogels did not induce any cytotoxicity by arresting RAW 264.7 macrophages in the G1 phase. Moreover, the significant increase in the proportion of cells in G2/M phase, observed after the 24-h interval, highlighted the proliferative effects of both types of nanohydrogels (GdDOTA⊂CS-TPP/HA and GdDOTP⊂CS-TPP/HA). These data were in agreement with those obtained by Avti et al., (2013) who noticed that Gd- containing single walled carbon nanotubes had no effect on G1 phase regulation of the NIH/3T3 mouse fibroblasts and did not lead to increases in the apoptotic cell population in the sub-G1 phase of the cell cycle [82].

The interaction of nanoparticles with plasma proteins (opsonins) and blood components (by hemolysis, thrombogenicity, and activation of the complement system) might influence their absorption and clearance and therefore affect their distribution and targeted delivery [83]. The size and surface charge of nanoparticles, which determine the interaction with plasma proteins and their absorption by macrophages, define their hemocompatibility profile [83]. Thus, electrically charged nanoparticles can induce hemolysis, thrombogenicity, and activation of the complement system [84], with a tendency to increase these effects proportional to the number of cationic groups attached to the surface [83].

Blood is the first contact tissue of organisms for nanoparticles (NPs) injected intravenously and the gateway of those administrated via other routes. Due to their dimensions, NPs traverse biological barriers, penetrate cells, and interact with blood components. In this context, the analysis of hemocompatibility of all NPs with therapeutic use is mandatory [85].

The cationic nature of chitosan as well as hyaluronic acid residues favors the interaction with the cellular components of the blood, which could have harmful effects in vivo. Alameh et al., showed that chitosan-based nanoparticles could induce hemolysis and hemagglutination in a dose-dependent manner [86]. In our experiments, the hemolysis threshold of 5% was not reached, suggesting that GdDOTA⊂CS-TPP/HA and GdDOTP⊂CS-TPP/HA were hemocompatible. These data are in line with those obtained by Jesus et al., which showed that none of chitosan nanoparticles and polymers induced a percentage of hemolysis superior to 5%, even at the chitosan concentration of 2.0 mg∙mL^−1^ [73]. 

On the other hand, the red blood cell surface presents negative charges due to the presence of sialic acids residues that could create repulsive electric forces between cells [87]. Taking in account that our NGs also have a negative exterior charge due to the hyaluronic acid residues, they could not contribute to the agglutination of erythrocytes.

In the case of nanomaterials given intravenously, complement proteins are the first barrier of immunity met by these in blood [88].

The complement system, consisting of 35 soluble proteins, is a crucial mediator of the innate and adaptive immune response and can be activated through proteolytic cleavages that are arranged in a cascade when a foreign body is detected.

Nanoparticle-mediated complement activation depends on the physicochemical characteristics of nanoparticles, such as chemical composition, zeta potential, size, and shape. Depending on their composition, NPs may induce complement activation through the classical, mannose-binding lectin, or alternative pathways [89]. Previously, it was proven that NPs between 40 and 250 nm induce an important activation of the complement system [90]. Our nanogels have dimensions in this size range and could activate the complement activation. However, this did not happen, probably due to their chitosan and hyaluronic acid contents. Marchand et al., hypothesized that chitosan, a positively charged biomaterial, binds to anionic plasma and serum proteins (C3, C5, factor B, Ba fragment, AT) without leading to complement activation [91].

In addition, HA, being a glycosaminoglycan that is present at the surface of many types of cells, could probably trick the immune system and the complement activation might not occur [92,93].

## 4. Materials and Methods

### 4.1. Materials

Sodium tripolyphosphate (TPP) was purchased from Acros Organics and sterile water from Laboratoire Aguettant, Lyon, France. 3-(4, 5-dimethylthiazol-2-yl)-2,5-diphenyl tetrazolium bromide (MTT), chitosan (CS, low viscosity from shrimp shells), sodium hyaluronate, sodium pyruvate, penicillin-streptomycin-amphotericin antibiotic mixture, hemoglobin from bovine blood, Triton X-100, mitochondrial membrane potential kit, and In Vitro Toxicology Assay Kit Lactic Dehydrogenase based were acquired from Sigma–Aldrich (St. Louis, MO, USA). Cell culture reagents and culture medium were provided by Gibco (Grand Island, NY, USA). The RAW 264.7 cell line was obtained from American Type Culture Collection (ATCC-TIB 71), Rockville, MD, USA. Complement MicroVue CH50 Eq Enzyme Immunoassay Kit was purchased from Quidel (Quidel, San Diego, CA, USA).

### 4.2. Synthesis and Characterizations of Nanohydrogels

Gd nanogel syntheses are described in previous papers [51,65,66]. Briefly, nanogels were obtained by an ionotropic gelation process. The polyanionic phase, constituted of HA (0.8 mg·mL^−1^) and TPP (1.2 mg·mL^−1^) in water (4.5 mL), was added dropwise to the CS solution (2.5 mg·mL^−1^ in 9 mL of a 10% (m/v) citric acid solution) under sonication (750 W, amplitude 32%) to obtain stable nanosuspensions. The gadolinium complex (GdDOTP or GdDOTA) was previously dissolved in the polyanion solution. At the end of addition, magnetic stirring was maintained for 10 min. Unloaded nanogels were obtained in the same way, omitting the gadolinium complexes. A dialysis step was conducted with a membrane of 25 kDa cut-off to remove unreacted compounds and to correct pH (3 cycles, against water for injection with a 1:100, *v*:*v* ratio). Nanosuspensions were then freeze-dried using glucose 15% (m/v) as a cryoprotectant. Nanoparticle average hydrodynamic diameters and polydispersity indexes were determined by dynamic light scattering (Malvern Zetasizer Nano-ZS, Malvern Instruments, Worcestershire, UK). Each nanosuspension was analyzed in triplicate at 20 °C at a scattering angle of 173°, after 1/20 dilution in water. Pure water was used as a reference dispersing medium. ζ-(zeta) potential data were collected through electrophoretic light scattering at 20 °C, 150 V, in triplicate for each sample, after 1/20 dilution in water. The instrument was calibrated with a Malvern –68 mV standard before each analysis cycle.

### 4.3. Biological Assay

#### 4.3.1. Cell Culture

The RAW 264.7 macrophage cells were cultured in DMEM (Dulbecco’s Modified Eagle Medium) medium containing 4 mM L-glutamine, 4.5 g/L glucose, and 1.5 g sodium bicarbonate supplemented with 1% PSA antibiotic (penicillin, streptomycin, and amphotericin) and 10% fetal bovine serum, pH 7.4. The culture medium was changed every 2–3 days, and the cells were harvested by scraping. RAW 264.7 cells were maintained in a humidity atmosphere (95%) with 5% CO_2_ at 37 °C and seeded in 24-well plates or 25 cm^2^ culture flasks according to the test applied. After reaching 80% confluence, the cell suspension was transferred to tubes and centrifuged at 1500 rpm for 5 min at 18 °C. The cell pellet was homogenized in complete DMEM medium, and cell counting was performed in the presence of 0.4% Tripan Blue using the Bürker Turk chamber.

Murine macrophages were treated with two types of nanogels, GdDOTA⊂CS-TPP/HA and GdDOTP⊂CS-TPP/HA, at doses of 2.5 μM, 5 μM, and 10 μM for 6 h and 24 h. Untreated cells were used as controls. For biological tests, the NGs lyophilized were dissolved with MilliQ ultrapure water at a final volume of 2 mL and before the experiments these were diluted in the culture medium in order to obtain the final doses mentioned above. The initial concentration of GdDOTA⊂CS-TPP/HA and GdDOTP⊂CS-TPP/HA was 9,8 × 10^−5^, respectively 2,4 × 10^−4^. The hemocompatibility tests were performed according to the Declaration of Helsinki and approved by the Ethics Committee of University of Bucharest. All subjects provided written informed consent to participate in this study which was approved by the institute’s ethics committee (No 48/26.04.2021).

#### 4.3.2. Cell Viability Assay

The cell viability of RAW 264.7 macrophages murine cells was assessed by the MTT test (3-(4, 5-dimethylthiazol-2-yl)-2,5-diphenyltetrazolium bromide) according to the method described by Mossman [94]. The cells were seeded in 24-well plates at a density of 5 × 10^4^ cells/mL and allowed to adhere for 24 h. These were then incubated with different doses of nanogels (2.5 μM, 5 μM, and 10 μM) for 6 and 24 h, respectively. After exposure, the culture medium was removed and 500 µL MTT (1 mg/mL) was added to each well. After two hours of incubation, the MTT solution was removed and the formazan crystals were solubilized with isopropanol 100%. The absorbance was measured spectrophotometrically at 595 nm using the FlexStation 3 Multi-Mode Microplate Reader (Molecular Devices LLC, San Jose, CA, USA).

#### 4.3.3. LDH Release Assay

The lactate dehydrogenase (LDH) activity was assessed using the In Vitro Toxicology Assay Kit (Sigma-Aldrich, St. Louis, MO, USA) according to the manufacturer’s instructions [95]. Briefly, the LDH release assay was measured using culture medium. The cells were seeded in 96-well plates and exposed to Gd NGs for 6 and 24 h. After these intervals, a volume of 50 μL of the sample (supernatant from each well) was treated with 100 μL reagent (cofactor, substrate, and dye) and incubated at room temperature for 30 min. The reaction was stopped with 1M HCl and the absorbance was measured spectrophotometrically at 450 nm using the FlexStation 3 Multi-Mode Microplate Reader (Molecular Devices LLC, San Jose, CA, USA).

#### 4.3.4. Mitochondria Membrane Potential

The mitochondria membrane potential (MMP) was evaluated using a mitochondrial membrane potential kit, MAK147, from Sigma-Aldrich (St. Louis, MO, USA). The method followed is based on the ability of a fluorescent cationic dye (JC-1) to accumulate in the mitochondria of healthy cells, resulting in an increase of the fluorescent signal. The RAW 264.7 cells were seeded at a density of 2 × 10^4^ cells/mL^−1^ in 96-well plates and incubated at 37 °C for 24 h. After that, the cells were treated with various doses of Gd nanogels (2.5 µM, 5 µM and 10 µM) for 6 and 24 h. The medium was removed and the cells were treated with 100 μL/well loading solution dye according to the instructions provided by the producer. After 30 min of incubation at 37 ° C, a volume of 50 μL/well Assay Buffer B was added. The fluorescence intensity was measured at 540 nm excitation and 590 nm emission using the FlexStation 3 Multi-Mode Microplate Reader (Molecular Devices LLC, San Jose, CA, USA) and SoftMax Pro software.

#### 4.3.5. Cell Cycle Analysis

RAW 267.4 cell cycle distribution was analyzed using flow cytometry after 6 h and 24 h of treatment with GdDOTA⊂CS-TPP/HA and GdDOTP⊂CS-TPP/HA NGs. An untreated sample served as control. Briefly, the cells were plated at an initial density of 10^6^ cells/25 cm^2^ and treated with the desired doses of GdDOTA⊂CS-TPP/HA and GdDOTP⊂CS-TPP/HA NGs (2.5 µM; 5 µM and 10 µM) in complete medium for 6 h and 24 h. At the end of each treatment time, cells were mechanically harvested from the culture surface, centrifuged, and washed with PBS buffer. Then, they were subsequently fixed for 15 min on ice, in cold ethanol and then washed in PBS buffer to remove residual ethanol. Fixed cells were resuspended in 100 µL RNase A (100 µg/mL) solution and incubated for 15 min at 37 °C. Next, 10 µL of Propidium iodide (PI) (100 µg/mL) were added in each sample for another 15 min right before the analysis on the flow cytometer.

Cell cycle progression of at least 15,000 events/sample was acquired in triplicate using a Beckman Coulter Gallios (Beckman Coulter, Indianapolis, IN, USA) flow cytometer and the Gallios Software (Indianapolis, IN, USA).

The data files generated were further analyzed for cell cycle distribution using Kaluza 1.5 Software (Indianapolis, IN, USA).

#### 4.3.6. Complement Activation

The complement cascade was measured using the MicroVue CH50 Eq Enzyme Immunoassay Kit (Quidel, San Diego, CA, USA) according to the manufacturer’s instructions. The total activity of complement in human serum samples was measured by quantifying the amount of terminal complexes (CBT) generated. Blood samples were taken from 10 potentially healthy volunteers (non-smokers, aged between 25 and 35 years, negative for HBV, HCV and HIV) with their consent, according to the ethical and sanitary norms [96]. Human blood was collected in tubes without anticoagulant and centrifuged at 1500× *g* (4 °C) for 10 min. Afterwards, 300 μL human serum were incubated with Gd NGs at various doses of 2.5 µM, 5 µM, and 10 µM, respectively, at 37 °C for 1 h. In the second step, the samples were incubated with an activator containing human γ globulins and mouse monoclonal antibodies in phosphate buffered saline (PBS) with 0.035% ProClin 300 at 37 °C for 60 min. During this time, the complement cascade is triggered and the terminal complexes are generated. Then, a volume of 100 µL sample with appropriate dilution was added into a 96 well plate treated with mouse monoclonal antibodies and was incubated at 15–30 °C for 60 min. In the third step, after being washed seven times (with a wash solution from the kit), 50 µL of conjugate sample were added, then incubated at 15–30 °C for 60 min. Finally, 100 µL of chromogenic substrate containing 3.3′, 5.5′ tetramethylenzidine (TMB) and hydrogen peroxide (H_2_O_2_) were added throughout the plate. The samples were incubated at 15–30 °C for another 15 min. Then, the reaction was stopped by the addition of 100 µL of hydrochloric acid 1 N, and the absorbance was measured spectrophotometrically at 450 nm using the FlexStation 3 Multi-Mode Microplate Reader (Molecular Devices LLC, San Jose, CA, USA). A human serum sample incubated at 37 °C served as a negative control. The results were calculated using the standard linear curve from the kit.

#### 4.3.7. Hemolytic Index

The hemolytic activity of lymphotropic nanogels was evaluated using erythrocytes from human venous blood according to the method described by Lu et al. [97]. Human blood was collected in tubes with 3.8% sodium citrate as an anticoagulant, and centrifuged at 1500× *g* (4 °C) for 10 min. The supernatant was removed, and the erythrocytes (RBC) were washed 4 times with 0.9% saline (1: 4 *v*/*v*) by centrifugation for 10 min at 1500× *g* (4 °C). Afterwards, the erythrocyte pellet was diluted in saline solution to a final concentration of 5% (*v*/*v*) and incubated with Gd NGs at doses of 2.5 µM, 5 µM, and 10 µM, respectively, at room temperature for 1, 6, and 24 h. After each time interval, samples were centrifuged for 1 min at 1500× *g* and a volume of 200 μL of supernatant was transferred to a 96-well plate (Nalgen Nunc International, NY, USA).

The amount of hemoglobin released into the supernatant was measured spectrophotometrically at 550 nm using the Appliskan Thermo Scientific reader (Termo-Fischer, Vantaa, Finland,). As a positive control, (100% hemolysis) Triton X-100 1% treated samples were used, and as negative control, (0% hemolysis) saline solution (0.9% NaCl) treated ones.

The percentage of hemolysis (% hemolysis) of erythrocytes was calculated with the following relation: Hemolysis (%) = (OD sample− OD negative control)/(OD positive control− OD negative control) × 100, where OD represent optical density read at 550 nm.

#### 4.3.8. The Thrombogenic Potential

The thrombogenic potential of nanogels was assessed by the method described by Pereira et al. [98]. As a first step, the coagulation reaction was initiated by adding of 10% 0.1 M CaCl_2_ to the whole blood.

The Gd nanogels doses of 2.5 µM, 5 µM, and 10 µM and 25% (*v*/*v*) PBS (negative control), respectively, were incubated with 150 µL of activated whole blood at room temperature for 0, 5, 15, 25, and 35 min in the 24 well plate. After each time interval, a volume of 3 mL of distilled water was added in each sample and these were incubated for 5 min at room’s temperature. In the presence of distilled water, the erythrocytes that were not trapped in the thrombus were lysed and thus hemoglobin was released. After incubation, a volume of 200 µL of the supernatant was transferred to a 96-well plate and the concentration of released hemoglobin was measured spectrophotometrically at 540 nm using the Apliskan plate reader (Termo-Fischer, Vantaa, Finland).

### 4.4. Statistical Analysis of Data

The statistical analyses were performed applying the student’s test (TTEST function, Microsoft Excel) or one-way ANOVA algorithm using GraphPad Prism Software (GraphPad software version 5.00, San Diego, CA, USA). The results obtained were expressed as the mean value of the triplicate experiments ± standard deviation (SD) (*n* = 3). The results are represented as the statistical significance (* = *p* <0.05, ** = *p* <0.01, *** = *p* <0.001). A value of *p* <0.05 was considered statistically significant. The data were plotted against the control, which was considered 100%.

## 5. Conclusions

In summary, our objective was to evaluate the systematic biosafety of nanogels incorporating GdDOTA and GdDOTP designed as new MRI contrast agents for the early diagnosis of tumors.

To this end, the effects of the GdDOTA⊂CS-TPP/HA and GdDOTP⊂CS-TPP/HA on RAW 264.7 murine macrophages in terms of metabolic viability, lactate dehydrogenase activity, as well as mitochondrial membrane potential and cell cycle distribution were evaluated. Meanwhile, for the human blood components, including red blood cells, complement and coagulation systems have been studied.

The in vitro studies showed that, in the given concentration range (up to 10 μM), GdDOTA⊂CS-TPP/HA and GdDOTP⊂CS-TPP/HA displayed non-toxic properties against RAW 264.7 macrophage cells. Moreover, both types of nanogels tested induced significant increases of RAW 267.4 cell proliferation after 24 h of incubation, with a significant dose dependent increase of G2/M cells being registered in the case of GdDOTA⊂CS-TPP/HA. The property of promoting cell proliferation could be due to the presence of biopolymers.

From the hemocompatibility point of view, our data indicated that both GdDOTA⊂CS-TPP/HA and GdDOTP⊂CS-TPP/HA present no risk of hemolysis (hemocompatible), had no effect on complement activation, and did not induce blood clotting (non-thrombogenic).

Our findings could be valuable for a better understanding of the advantages of the encapsulation of chelates, such as GdDOTA and GdDOTP, into a biocompatible matrix containing chitosan and hyaluronic acid for developing new, safe, and effective contrast agents for the magnetic resonance imaging of lymph nodes.

## Figures and Tables

**Table 1:** Physico-chemical properties of the purified nanogels.

	GdDOTA⊂CS-TPP/HA	GdDOTP⊂CS-TPP/HA
D_H_ (nm)	217	242
PdI	0.22	0.22
ζ (mV)	30.3	31.9
[Gd]_NS_ (M)	0.98 × 10^−4^	2.4 × 10^−4^

Diameter hydrodynamic D_H_ (nm), polydispersity index (PdI), zeta potential ζ (mV) and gadolinium concentrations [Gd]_NS_ (M).

**Table 2:** The thrombogenic potential exposed at various doses (2.5, 5 and 10 μM) of GdDOTA⊂CS-TPP/HA and GdDOTP⊂CS-TPP/HA NGs.

Samples		Time (Minutes)			
		5 Min	15 Min	25 Min	35 Min
Control		1.507 ± 1.98	0.636 ± 2.03	0.172 ± 5.16	0.103 ± 4.67
GdDOTA⊂CS-TPP/HA NGs	2.5 µM	1.531 ± 3.12	0.664 ± 5.11	0.182 ± 1.86	0.103 ± 2.05
	5 µM	1.469 ± 2.37	0.629 ± 1.11	0.185 ± 8.82	0.107 ± 9.7
	10 µM	1.510 ± 1.26	0.651 ± 3.78	0.145 ± 4.07	0.092 ± 1.94
GdDOTP⊂CS-TPP/HA NGs	2.5 µM	1.511 ± 2.02	0.625 ± 3.83	0.173 ± 4.4	0.105 ± 2.66
	5 µM	1.470 ± 1.4	0.61 ± 1.72	0.167 ± 2.24	0.101 ± 2.42
	10 µM	1.373 ± 1.99	0.645 ± 1.84	0.148 ± 9.25	0.098 ± 5.32

PBS was used as a negative control. Data are expressed as mean ± SD (*n* = 3).

## Data Availability

Not applicable.
